# Preparation of Herbal Formulation for Inflammatory Bowel Disease Based on In Vitro Screening and In Vivo Evaluation in a Mouse Model of Experimental Colitis

**DOI:** 10.3390/molecules24030464

**Published:** 2019-01-28

**Authors:** Jaemin Lee, Han-Seok Choi, Jinkyung Lee, Jimin Park, Sang-Back Kim, Myoung-Sook Shin, Sullim Lee, Gwi Seo Hwang, Bon Am Koo, Ki Sung Kang

**Affiliations:** 1 College of Korean Medicine, Gachon University, Seongnam-si, Gyeonggi-do 13120, Korea; jaemin.lee426@gmail.com (J.L.); jklee9441@hanmail.net (J.L.); ms.shin@gachon.ac.kr (M.-S.S.); seoul@gachon.ac.kr (G.S.H.); 2 New Drug Research Team, Kolmar, Korea Co. Ltd., Sandan-gil, Jeonui-myeon, Sejong-si 30003, Korea; fcosmos@kolmar.co.kr (H.-S.C.); jimpark@kolmar.co.kr (J.P.); m302@kolmar.co.kr (S.-B.K.); 3 College of Bio-Nanotechnology, Gachon University, Seongnam-si, Gyeonggi-do 13120, Korea; sullimlee@gachon.ac.kr

**Keywords:** inflammatory bowel disease, anti-inflammatory activity, *Zingiber officinale*, *Terminalia chebula*, *Aucklandia lappa*

## Abstract

Many medicinal plants have been used traditionally in East Asia for the treatment of gastrointestinal disease and inflammation. The aim of this study was to evaluate the anti-inflammatory activity of 350 extracts (175 water extracts and 175 ethanol extracts) from 71 single plants, 97 mixtures of two plants, and seven formulations based on traditional medicine, to find herbal formulations to treat inflammatory bowel disease (IBD). In the in vitro screening, nitric oxide (NO), tumor necrosis factor (TNF)-α, and interleukin (IL)-6 levels were determined in LPS-treated RAW264.7 cells and the TNF-α induced monocyte-epithelial cell adhesion assay was used for the evaluation of the anti-inflammatory activity of the compounds. Dextran sulfate sodium (DSS)-induced colitis model and 2,4,6-trinitrobenzene sulfonic acid (TNBS)-induced colitis model were used to evaluate the therapeutic effect against IBD of the samples selected from the in vitro screening. KM1608, composed of *Zingiber officinale, Terminalia chebula* and *Aucklandia lappa*, was prepared based on the screening experiments. The oral administration of KM1608 significantly attenuated the severity of colitis symptoms, such as weight loss, diarrhea, and rectal bleeding, in TNBS-induced colitis. In addition, inflammatory mediators, such as myeloperoxidase, TNF-α, and IL-6 levels decreased in the lysate of colon tissues treated with KM1608. Collectively, KM1608 ameliorated colitis through the regulation of inflammatory responses within the colon, which indicated that KM1608 had potential for the treatment of IBD.

## 1. Introduction

Inflammatory bowel disease (IBD) is an idiopathic chronic inflammatory condition of the gastrointestinal tract, comprising Crohn’s disease and ulcerative colitis. The symptoms of IBD are chronic diarrhea, abdominal pain, rectal bleeding, weight loss, and shortening of the colon. Although the etiology of IBD remains uncertain, it is known that irregular immune response, gut microbial flora, and genetic and environmental factors are associated with the pathogenesis of IBD [1,2]. The present treatment regimen, including aminosalicylates, corticosteroids, biologics, and immunosuppressants, has therapeutic limits and leads to side effects [3]. Furthermore, recent failures of drug targets in IBD, such as IL-17, IL-13, interferon (IFN)-γ, and chemokine receptor (CCR)-9, have indicated that single target therapy for IBD is difficult owing to pathogenic heterogeneity [4]. Therefore, the discovery of alternative treatment options with multiple therapeutic targets is required. We expect that natural product formulations, such as medicinal plant extracts or traditional medicines, would offer excellent alternative therapies for IBD.

In recent decades, medicinal plant extracts, traditional medicines and their active components have been investigated for the development of novel anti-inflammatory drugs [5,6,7,8,9,10]. Many patients with IBD are interested in alternative treatments because they are dissatisfied with the current conventional treatment [11]. However, no successful therapeutics for IBD based on natural products have been developed. Thus, we aimed to explore potent herbal formulations for the treatment of IBD by using a large-scale screening test.

We tested the anti-inflammatory activity of 350 samples that were extracted (in water and 50% ethanol) from 71 single plants, 97 mixtures of two plants, and seven formulations based on traditional medicine. These plant and formulation samples were selected from various sources of traditional Chinese medicine literature, such as *Shanghan Lun*, *Compendium of Materia Medica*, and *Traditional Chinese Medicine Formulary.* In the in vitro screening, we determined NO production and the levels of pro-inflammatory cytokines (TNF-α and IL-6) in RAW264.7 cells, and assayed the monocyte (U937)-epithelial (HT-29) adhesion ability. In the in vivo screening, we examined the therapeutic efficacy of the selected samples in mouse models of DSS-induced colitis and TNBS-induced colitis. Furthermore, we also investigated the effects of KM1608, the final formulation selected, on TNBS-induced colitis.

## 2. Results and Discussion

### 2.1. NO Assay for Preliminary Screening of Plant Extracts

First, we screened both the water and the 50% ethanol extracts of 71 samples of single plants (Table 1), 97 samples of a 1:1 mixture of two plants (Table 2), and seven samples of a formulation based on traditional medicine (Table 3). 

Nitric oxide (NO) is synthesized from l-arginine by nitric oxide synthases (NOSs), such as endothelial NOS (eNOS), constitutive NOS (cNOS), and inducible NOS (iNOS), in various cells. In the patients with IBD, NO production and iNOS activity were increased in the inflamed colonic mucosa [12]. Thus, to evaluate anti-inflammatory effect of each sample (100 μg/mL), we performed the NO assay on RAW264.7 cells stimulated with LPS (1 μg/mL). Samples that were extracted with 50% ethanol tended to inhibit NO production more than samples extracted with water (Table 1, Table 2 and Table 3). In this assay, 72 extracts, which resulted in a decrease of more than 80% in NO production, were selected for the subsequent monocyte adhesion assay.

### 2.2. TNF-α Induced Monocyte-Epithelial Cell Adhesion Assay

The adhesion of inflammatory cells to colonic epithelial cells is an important event in colonic inflammation. In the colonic mucosa, immune cells, such as macrophages and T lymphocytes, infiltrate the mucosal layer and are located in close proximity to the epithelial layer in inflammatory conditions such as IBD. The interaction between immune cells and epithelial cells releases inflammatory mediators, such as NO, TNF-α, and IL-6, and ultimately results in the disruption of the barrier function of the intestinal epithelium [13]. Therefore, we performed a TNF-α-induced monocyte-epithelial cell adhesion assay to screen the 72 selected extracts (at 100 μg/mL) using HT-29 as the epithelial cell line and U937 as the monocyte cell line, which are precursors of macrophages. U937 cells were prelabeled with 2′,7′-bis-(2-carboxyethyl)-5-(and-6)-carboxyfluorescein/acetoxy-methyl ester (BCECF/AM) for 30 min before co-incubation with HT-29 cells. After co-incubation for 30 min, the wells were washed to remove unadhered cells, and the BCECF fluorescence was measured to evaluate cell-to-cell adhesion. 5-aminosalicylic acid (5-ASA, 20 mM) was used as the reference drug. In this assay, we selected 27 extracts that resulted in 80% lower BCECF fluorescence than TNF-α (100 ng/mL) alone (Table 4).

### 2.3. TNF-α and IL-6 Production in RAW264.7 Cells

**Figure 1:**
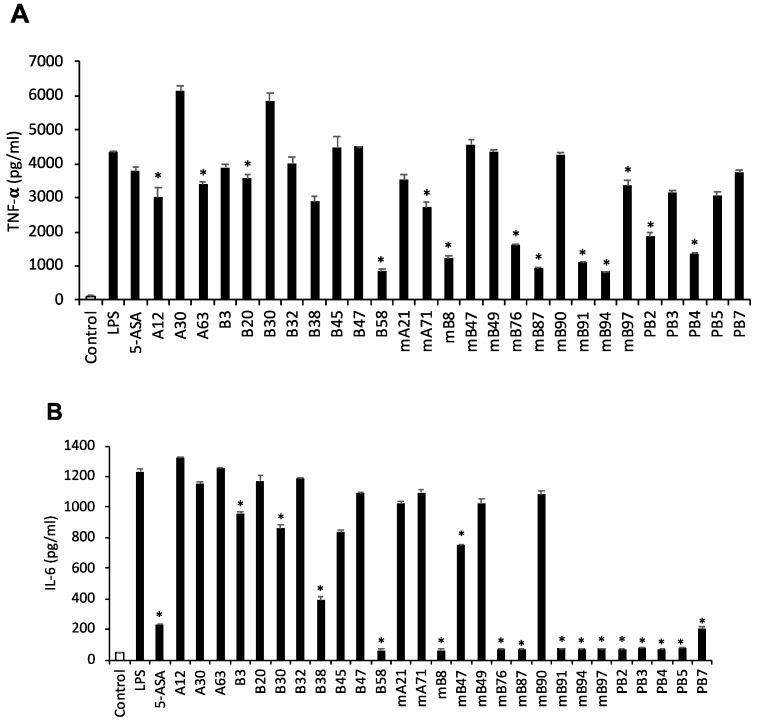
Effect of selected 27 samples on TNF-α and IL-6 production in LPS-stimulated RAW264.7 cells. RAW264.7 cells (3 × 10^5^ cells/well, 24-well plate) were first treated with LPS (1 μg/mL) for 1 h and then treated with 5-ASA (20 mM) or sample (100 μg/mL) for 24 h. TNF-α and IL-6 concentrations were measured by using ELISA (**A** and **B**). Sample labels were following: <Water extracts> A12 (*Pulsatilla koreana*), A30 (*Melia azedarach*), A63 (*Cinnamomum cassia*), mA21(*Myristica fragrans* and *Sanguisorba hakusanensis*), mA71 (*Rheum palmatum* and *Zingiber officinale*), <Ethanol extracts> B3 (*Zingiber officinale*), B20 (*Bupleurum falcatum*), B30 (*Melia azedarach*), B32 (*Patrinia scabiosaefolia*), B38 (*Jeffersonia dubia*), B45 (*Machilus thunbergii*), B47 (*Terminalia chebula*), B58 (*Aucklandia lappa*), mB8 (*Paeonia lactiflora* and *Aucklandia lappa*), mB47 (*Sanguisorba hakusanensis* and *Zingiber officinale*), mB49 (*Sanguisorba hakusanensis* and *Euryale ferox*), mB76 (*Rheum palmatum* and *Aucklandia lappa*), mB87 (*Zingiber officinale* and *Aucklandia lappa*), mB90 (*Terminalia chebula* and *Machilus thunbergii*), mB91 (*Terminalia chebula* and *Aucklandia lappa*), mB94 (*Euryale ferox and Aucklandia lappa*), mB97 (*Machilus thunbergii* and *Aucklandia lappa*), PBs (prescriptions, see Table 3). The control group was treated with 0.1% DMSO. The data are presented as the mean ± SEM of three independent experiments. * *p* < 0.05 vs. LPS.

We determined the levels of inflammatory cytokines (TNF-α and IL-6) in LPS-stimulated RAW264.7 cells to evaluate the anti-inflammatory profile of the selected samples. 5-ASA was used as the reference drug. First, RAW264.7 cells were treated with LPS (1 μg/mL) for 1 h, and then samples (at 100 μg/mL) or 5-ASA (20 mM) were added for 24 h. In this assay, we selected seven extract samples (B58, mB8, mB76, mB87, mB91, mB94, and mB97; Figure 1) that resulted in significant inhibition of the production of both cytokines. Therefore, we screened these seven samples in vivo.

### 2.4. In Vivo Screening of Seven Samples in Mouse Model of Experimental Colitis

**Figure 2:**
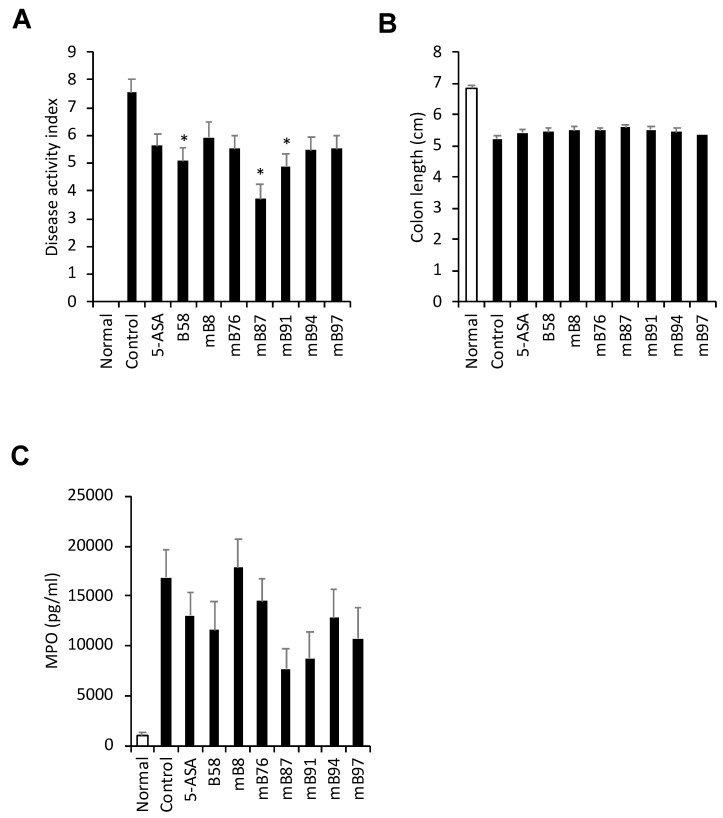
Effect of seven selected samples on DSS-induced colitis in mice. Colitis was induced in C57BL/6 mice by the administration of 1.7% DSS in the drinking water for 7 days. The animals were orally administered the extract samples (200 mg/kg) or 5-ASA (200 mg/kg), the reference drug, once per day. The disease activity index was scored during the experiment (**A**). Colon length was measured at necropsy (**B**). MPO in the colon lysate was measured by using an ELISA kit (**C**). The data are presented as the mean ± SEM. n = 6–8, * *p* < 0.05 vs. control.

**Figure 3:**
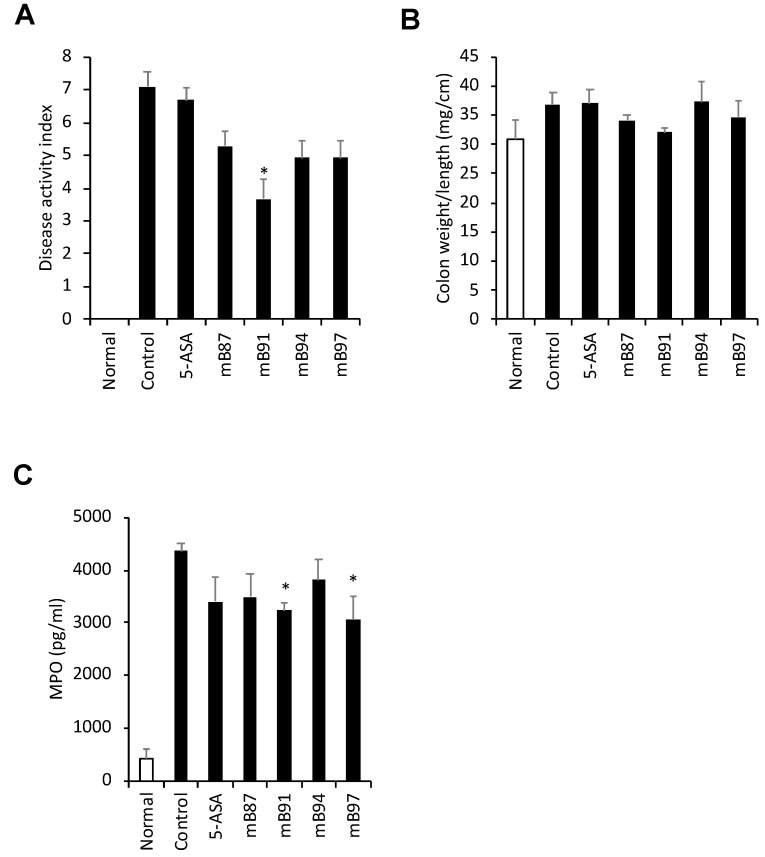
Effect of four selected samples on TNBS-induced colitis in mice. Colitis was induced in ICR mice by the intrarectal injection of TNBS. The animals were treated with extract samples (200 mg/kg) or 5-ASA (200 mg/kg), used as the reference drug, once per day. Disease activity index was scored during the experiment (**A**). Colon weight and length were measured at necropsy (**B**). MPO in the colon tissue lysate was measured by using an ELISA kit (**C**). The data are presented as the mean ± SEM. n = 6–8, * *p* < 0.05 vs. control.

In the in vivo screening, we employed C57BL/6 mice with DSS-induced colitis for the seven selected samples (B58, mB8, mB76, mB87, mB91, mB94, and mB97). 5-ASA (200 mg/kg) and all samples (200 mg/kg) were orally administered once per day during the DSS-administration period. In the DSS-induced colitis model, we used the indices of disease activity, colon length, and myeloperoxidase (MPO) activity to evaluate the efficacy of samples. Samples B58, mB87, and mB91 significantly ameliorated the symptoms of colitis, and 5-ASA and other samples tended to ameliorate the disease activity index (DAI) (Figure 2A). MPO, abundantly expressed in neutrophils, was measured to determine the levels of inflammation. 5-ASA and all samples except for mB8, slightly decreased MPO activity in the colon tissue (Figure 2C). Samples mB87, mB91, mB94, and mB97 tended to improve DSS-induced colitis; therefore, we further investigated the efficacy of these samples in ICR mice with TNBS-induced colitis. Although a single plant sample, B58, exerted an ameliorative effect of colitis, it was omitted from the next step because it was included in all of the mixture samples. In the TNBS-induced colitis model, we used the indices of disease activity, colon weight/length ratio, and MPO activity to evaluate efficacy. Sample mB91 resulted in a significant improvement in colitis and other samples led to a slight improvement in colitis in the DAI (Figure 3A). The colon weight/length ratio was measured as an index of edema in the inflamed colon. Samples mB87 and mB91 slightly decreased the colon weight/length ratio (Figure 3B). 5-ASA and all samples slightly decreased MPO activity in the colon tissue (Figure 3C). Sample mB87 (*Zingiber officinale* and *Aucklandia lappa)* and mB91 (*Terminalia chebula* and *Aucklandia lappa*) resulted in a significant improvement in both types of experimental colitis. From these results, we decided to prepare a formulation of the three medicinal plants based on mB87 and mB91, called KM1608 (a 1:2:2 mixture of *Zingiber officinale*, *Terminalia chebula*, and *Aucklandia lappa*).

### 2.5. Effect of KM1608 on Mice with TNBS-Induced Colitis

**Figure 4:**
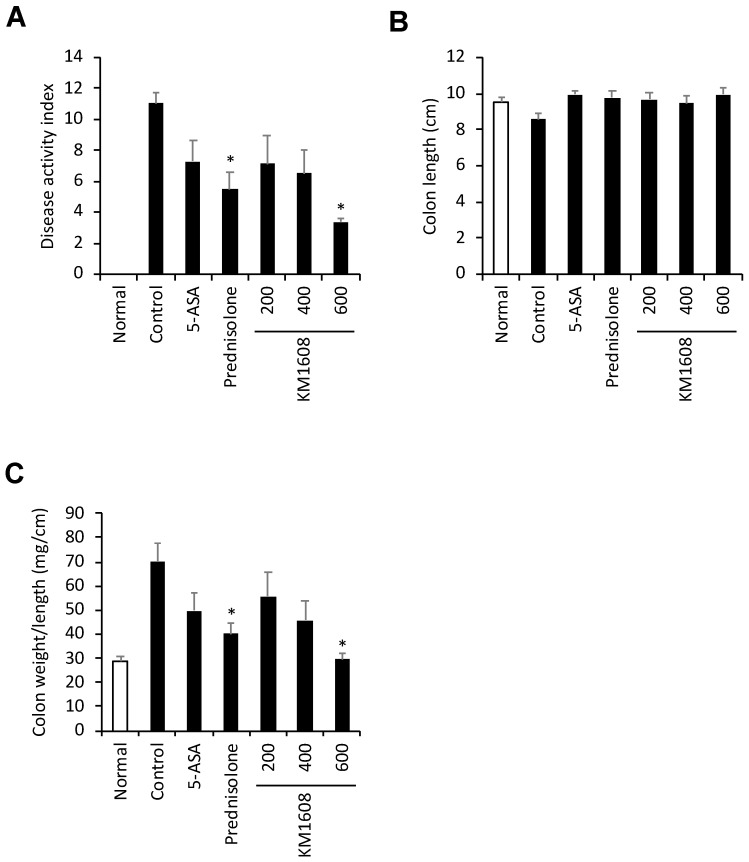
Effect of KM1608 on TNBS-induced colitis in mice. Colitis was induced in ICR mice by the intrarectal injection of TNBS. The animals were treated with KM1608 (200, 400, and 600 mg/kg), 5-ASA (200 mg/kg), and prednisolone (5 mg/kg) orally once per day. 5-ASA and prednisolone were used as the reference drugs. The disease activity index was scored during the experiment (**A**). Colon length and weight were measured at necropsy (**B**,**C**). The data are presented as the mean ± SEM. n = 6–8, * *p* < 0.05 vs. control.

**Figure 5:**
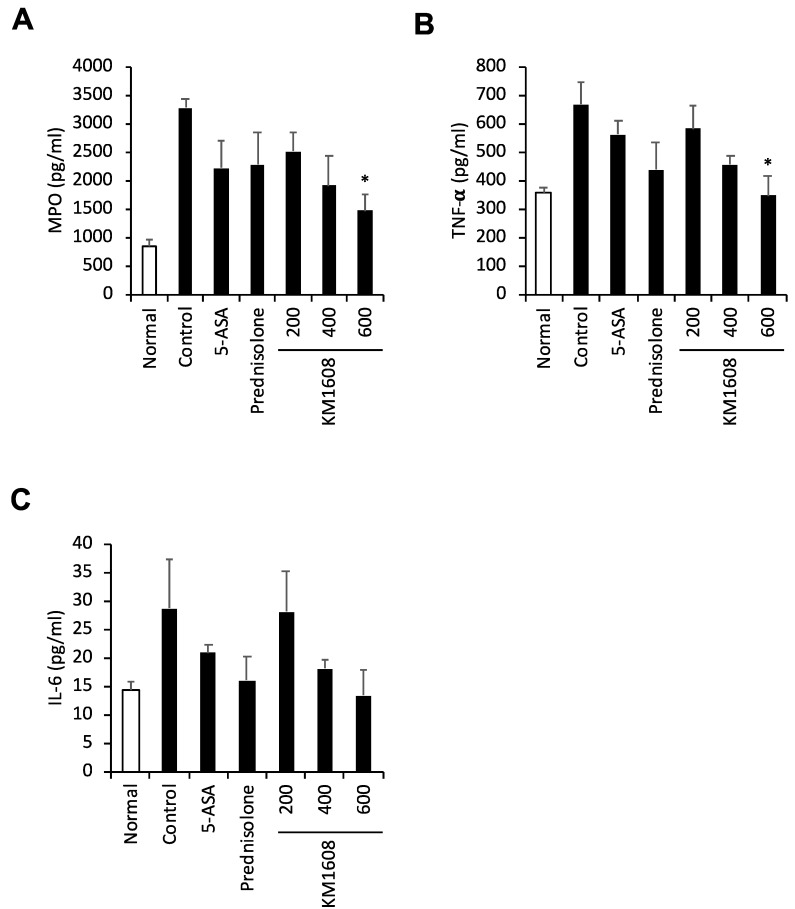
KM1608 inhibits the inflammatory factors involved in TNBS-induced colitis. Colitis was induced in ICR mice by the intrarectal injection of TNBS. The animals were treated with KM1608 (200, 400, and 600 mg/kg), 5-ASA (200 mg/kg), and prednisolone (5 mg/kg) orally once per day. 5-ASA and prednisolone were used as the reference drugs. The MPO, TNF-α, and IL-6 levels in the colon tissue lysates were measured by using ELISA kits (**A**–**C**). The data are presented as the mean ± SEM of three independent experiments. * *p* < 0.05 vs. control.

We conducted a more detailed investigation of the in vivo efficacy of KM1608 in TNBS-induced colitis. KM1608 (200, 400, and 600 mg/kg), 5-ASA (200 mg/kg), and prednisolone (5 mg/kg) were orally administered once per day. 5-ASA and prednisolone were used as the reference drugs. We used the following indices to evaluate the efficacy of KM1608: DAI, colon length, colon weight/length ratio, MPO activity, TNF-α, and IL-6. KM1608 significantly decreased the DAI and the colon weight/length ratio in a dose-dependent manner (Figure 4A,C). KM1608 resulted in a slight improvement in the colon length (Figure 4B). We determined the MPO activity and pro-inflammatory cytokines (TNF-α and IL-6) as a marker of inflammation in the colon tissue of colitis-induced mice. KM1608 (600 mg/kg) administration resulted in a significant decrease in MPO activity and TNF-α level (Figure 5A,B), and slightly decreased IL-6 level in the colon tissue lysate (Figure 5C). In addition, KM1608 (600 mg/kg) resulted in better parameters for many of the indices used for colitis evaluation than 5-ASA and prednisolone.

Medicinal plants generally contain multiple bioactive compounds that are responsible for the beneficial effects on human diseases through synergistic actions [14]. Synergistic anti-inflammatory effects of the compound combination of the herbal formula GuGe FengTong, prepared from three herbs, *Spatholobus suberectus* (Leguminosae), *Dioscorea nipponica* (Dioscoreaceae), and *Zingiber officinale* (Zingiberaceae), were recently reported [15]. Compared with the single compounds, the combination of two compounds, biochanin A and 6-gingerol, could synergistically inhibit the production of pro-inflammatory cytokines (TNF-α, IL-1β, and IL-6) and the activation of MAPK signaling pathway in LPS-stimulated RAW264.7 cells [15]. In addition, a combination effect was also evident on the antioxidant and anti-inflammatory effects of honokiol and modified citrus pectin in mouse monocytes [16]. In other research areas, synergistic antidiabetic activity of *Vernonia amygdalina* and *Azadirachta indica* has also been reported [17]. Synergism has also supported and been identified in drug-target interaction studies, in which drugs with multiple target network mechanisms are believed to have greater efficacy for the treatment of disease [18].

Several studies have reported the anti-inflammatory effect of plant extracts; for example, *Terminalia chebula* and *Zingiber officinale* ameliorated acetic acid-induced colitis in rats via the inhibition of MPO activity [19] and the inhibition of MPO activity, TNF-α, prostaglandin E2 [20], respectively, and *Aucklandia lappa* extract ameliorated DSS-induced colitis in mice via the inhibition of IFN-γ and IL-6 [21]. Moreover, 6-gingerol, active component of *Zingiber officinale*, has anti-inflammatory activity via inhibiting NO production, iNOS expression and cyclooxygenase activity [22], ellagic acid, active component of *Terminalia chebula*, inhibits cyclooxygenase activity and reduces paw edema in the carrageenan-induced edema [23], and also dehydrocostus lactone, active component of *Aucklandia lappa*, inhibits NO and TNF-α production in LPS-activated RAW264.7 cells [24]. In the present study, the administration of KM1608, a mixture of the extracts from three plants, exerted more potent therapeutic effects than the administration of each plant individually and the mixture of the extracts of two plants at the same dose in DSS-induced colitis. These results indicate that KM1608 has therapeutic potential for the treatment of IBD.

## 3. Materials and Methods

### 3.1. Plant Material

All medicinal plant samples were purchased from Songrim Muyak (Seoul, Korea). The samples were extracted twice with water or 50% ethanol (*v*/*v*) at 80 °C for 3 h, and the extracted solutions were filtered and evaporated. The samples that consisted of a mixture of two plants were mixed at a 1:1 (*w*/*w*) ratio, traditional medicine formulations were mixed at the ratios based on prescription, and KM1608 was mixed in a 1:2:2 ratio (*Zingiber officinale*: *Terminalia chebula*: *Aucklandia lappa*) before the extraction process. The extracts were then freeze dried to obtain the powders used as the test extract samples.

### 3.2. Animal and Cell Culture

Seven-week-old female C57BL/6 and ICR mice were purchased from Daehan Bio Link (Seoul, Korea) and acclimated for 7 days in a specific pathogen-free (SPF) environment under constant conditions (temperature: 23 °C ± 2 °C; humidity: 50% ± 5%; light/dark cycle: 12 h) at a facility in Kolmar Korea Co., Ltd. (Sejong, Korea). All animal studies were performed in accordance with the instructions of the Ethics Committee for Use of Experimental Animals at Kolmar Korea Co., Ltd. (confirmation number: 16-NP-IBD-011-P). The RAW264.7 mouse macrophage cell line was purchased from the ATCC (Manassas, VA, USA), and the cells were seeded in DMEM supplemented with 10% heat-inactivated FBS and 1% penicillin-streptomycin obtained from Life Technologies (Waltham, MA, USA). The human colorectal adenocarcinoma cell line, HT-29, and the human monocytic cell line, U937, were purchased from the ATCC (USA) and seeded in RPMI supplemented with 10% heat-inactivated FBS and 1% penicillin-streptomycin. The cells were maintained at 37 °C in a humidified atmosphere containing 5% CO_2_.

### 3.3. Determination of Nitric oxide (NO), TNF-α, IL-6, and MPO Production

RAW264.7 cells (3 × 10^5^ cells/well in a 24-well plate) were treated LPS (1 μg/mL) for 1 h and then treated with 5-ASA (20 mM) or samples (100 μg/mL) for 24 h. After incubation for 24 h, nitrite production was estimated by using Griess reagent [25] and a standard curve previously prepared using sodium nitrite (Promega, Fitchburg, WI, USA). The cell supernatant (50 μL) was mixed with an equal volume of Griess reagent and the absorbance at 540 nm was measured by using a microplate reader (Molecular Devices Co., San Jose, CA, USA). For the analysis of IL-6, TNF-α, and MPO in colitis-induced colon tissue, colon tissue samples were suspended in lysis buffer (Intron, Seoul, Korea) and ground by using a homogenizer (Scilogex, Rocky Hill, CT, USA). The supernatant was collected by centrifugation (10,000 rpm, 20 min, 4 °C). The IL-6, TNF-α (R&D Systems, Minneapolis, MN, USA), and MPO (HK210, Hycult Biotechnology, Wayne, PA, USA) levels in the supernatant were measured by using ELISA kits in accordance with the manufacturer’s instructions.

### 3.4. Monocyte Adhesion Assay

U937 cells were prelabeled with BCECF/AM (10 μg/mL, Sigma, St. Louis, MO, USA) for 30 min at 37 °C. HT-29 cells (2 × 10^6^ cells/well in 48-well plates)) were pretreated with sample (100 μg/mL) or 5-ASA (20 mM) for 1 h and then stimulated with TNF-α for 24 h [26]. Subsequently, HT-29 cells were co-incubated with BCECF/AM-prelabeled U937 cells (5 × 10^5^ cells/well) for 30 min at 37 °C. The wells were washed twice with PBS to remove unadhered U937 cells. The cells were lysed with 0.1% Triton X-100 in 0.1 M Tris and BCECF fluorescence was analyzed by using a microplate reader (TECAN, Grödig, Austria), with excitation at 485 nm and emission at 520 nm.

### 3.5. DSS-Induced Colitis

Acute colitis was induced in C57BL/6 mice for 7 days by the addition of 1.7% (*w*/*v*) DSS to drinking water. Daily measurements of body weight, stool consistency, and rectal bleeding were conducted. The normal group received water without DSS. The control group received drinking water containing 1.7% DSS. The sample groups received DSS-containing drinking water and extract samples (200, 400, or 600 mg/kg). The 5-ASA group received DSS-containing drinking water and 5-ASA (200 mg/kg). The prednisolone group received DSS-containing drinking water and prednisolone (5 mg/kg). Carboxymethylcellulose (CMC) solution (0.5%) was used to dissolve the extract samples, 5-ASA, and prednisolone for in vivo experiments. All drugs were orally administered once per day during the experiment. A clinical assessment was performed to determine the DAI. DAI comprised the total score of each of the following: (weight loss: 1 = 1–5%; 2 = 5–10%; 3 = 10–20%; 4 = >20%; stool consistency: 0 = normal; 2 = loose stool; 4 = diarrhea; and rectal bleeding: 0 = negative; 2 = mild; 4 = severe). If an animal died, DAI was scored as 15. The animals were sacrificed after 7 days of DSS treatment, and the colon length was measured.

### 3.6. TNBS-Induced Colitis

Acute colitis was induced in ICR mice. A 100 μL aliquot of 0.5% TNBS solution dissolved in ethanol (50%, *v*/*v*) was instilled into the colon via a cannula to induce colitis. To prevent outflow of the agents from the anus, mice were held in the head-down position for 1 min after the instillation. Body weight and disease symptoms were assessed on three subsequent days. A clinical assessment was performed to determine the DAI. DAI was scored as described in Section 3.5. If an animal died, DAI was scored as 15. After 5 days of TNBS injection, the animals were sacrificed and then colon edema, length, and weight were measured.

### 3.7. Statistical Analysis

The results are expressed as the mean ± SEM. Statistical comparisons were performed by using one-way analysis of variance (ANOVA) followed by Tukey’s test. A value of *p* < 0.05 was considered to indicate significant difference.

## 4. Conclusions

This study was conducted to discover potent formulations of natural products with anti-inflammatory activity. In the in vitro screening experiments, we tested the effects of 350 extracted samples on NO, TNF-α, and IL-6 production in LPS-stimulated RAW264.7 cells and on TNF-α-induced monocyte (U937)-colonic epithelial (HT-29) adhesion ability. Finally, we found the formulation KM1608, composed of *Zingiber officinale*, *Terminalia chebula*, and *Aucklandia lappa*, through in vivo screening in a mouse model of experimental colitis. KM1608 significantly ameliorated the severity of colitis and the colon weight/length ratio in a dose-dependent manner. In addition, KM1608 inhibited MPO activity and pro-inflammatory cytokines in the colon tissue lysate of DSS-induced colitis. Moreover, the ameliorative effect of KM1608 on DSS-induced colitis was more potent than that of 5-ASA or prednisolone. Collectively, KM1608 administration improved the symptoms of colitis and the inflammatory responses. IBD such as ulcerative colitis and Crohn’s disease are very complicate disease to control pathophysiology with single target therapy. Many trials have failed to development of IBD therapeutics with single target agents. We believe that future studies are required to consider multiple target therapy and/or synergistic combination like as herbal formulation. We hope that our present study and the formulation will provide useful ways for the treatment of IBD.

## Figures and Tables

**Table 1:** NO assay results of single plant extracts.

No.	Plant	% of LPS	
		Water Extract (Group A)	Ethanol Extract (Group B)
	Control (0.1% DMSO without LPS)	18.9 ± 0.1	20.0 ± 0.6
1	*Glycyrrhiza uralensis*	100.3 ± 3.1	102.3 ± 2.4
2	*Brassica juncea*	99.2 ± 1.5	97.2 ± 1.0
3	*Zingiber officinale*	99.7 ± 3.8	68.1 ± 1.7
4	*Sophora flavescens*	101.5 ± 1.2	102.8 ± 1.8
5	*Tussilago farfara*	98.5 ± 5.6	99.6 ±2.0
6	*Codonopsis pilosula*	90.4 ± 2.6	100.2 ± 2.6
7	*Ephedra sinica*	95.8 ± 5.2	100.5 ± 0.8
8	*Paeonia suffruticosa*	104.1 ± 7.1	106.5 ± 5.1
9	*Inula helenium*	90.2 ± 3.8	86.3 ± 2.6
10	*Pinellia ternata*	93.1 ± 3.6	96.0 ± 3.7
11	*Saposhnikovia divaricata*	99.8 ± 4.5	99.7 ± 2.1
12	*Pulsatilla koreana*	64.2 ± 2.8	60.5 ± 2.3
13	*Atractylodes macrocephala*	96.3 ± 3.8	94.3 ± 2.5
14	*Psoralea corylifolia*	94.8 ± 2.6	98.9 ± 2.6
15	*Belamcanda chinensis*	95.9 ± 2.6	101.0 ± 2.8
16	*Dioscorea batatas*	97.9 ± 1.7	102.1 ± 2.8
17	*Phytolacca esculenta*	98.2 ± 35	106.1 ± 4.2
18	*Acorus gramineus*	95.9 ± 5.1	102.3 ± 0.9
19	*Asiasarum sieboldi*	103.7 ± 2.9	106.0 ± 2.7
20	*Bupleurum falcatum*	97.2 ± 2.6	70.1 ± 0.5
21	*Magnolia kobus*	114.2 ± 2.2	111.5 ± 2.7
22	*Achyranthes bidentata*	93.1 ± 4.9	97.5 ± 1.7
23	*Daphne genkwa*	95.1 ± 3.8	89.8 ± 2.5
24	*Myristica fragrans*	108.1 ± 2.5	109.3 ± 1.3
25	*Coix lachryma-jobi*	90.8 ± 7.5	104.1 ± 0.4
26	*Aster tataricus*	94.4 ± 2.2	99.9 ± 4.1
27	*Paeonia lactiflora*	96.2 ± 1.7	97.8 ± 3.1
28	*Citrus unshiu*	102.3 ± 3.1	108.0 ± 2.5
29	*Cnidium officinale*	104.1 ± 2.8	105.3 ± 2.3
30	*Melia azedarach*	47.8 ± 0.7	46.5 ± 1.6
31	*Morinda citrifolia*	102.4 ± 4.3	100.0 ± 1.8
32	*Patrinia scabiosaefolia*	90.2 ± 2.5	67.3 ± 2.7
33	*Prunella vulgaris*	76.4 ± 1.4	74.4 ± 3.3
34	*Prunus armeniaca*	104.7 ± 2.8	98.7 ± 2.1
35	*Corydalis remota*	109.3 ± 3.8	100.2 ± 2.0
36	*Scutellaria baicalenesis*	39.3 ± 0.2	35.1 ± 1.6
37	*Astragalus membranaceus*	101.9 ± 1.9	96.9 ± 2.1
38	*Jeffersonia dubia*	117.0 ± 5.1	70.1 ± 2.5
39	*Rumex japonicus*	113.8 ± 4.0	108.0 ± 2.2
40	*Smilax china*	107.4 ± 3.9	101.9 ± 2.0
41	*Elsholtzia ciliata*	93.0 ± 0.9	99.5 ± 1.6
42	*Angelica gigas*	104.3 ± 7.1	92.9 ± 1.8
43	*Evodia officinalis*	97.8 ± 3.0	101.0 ± 10.9
44	*Aconitum carmichaeli*	103.0 ± 4.8	106.1 ± 7.1
45	*Machilus thunbergii*	100.3 ± 5.4	28.9 ± 3.0
46	*Atractylodes japonica*	104.9 ± 11.2	90.4 ± 2.9
47	*Terminalia chebula*	58.3 ± 4.9	47.9 ± 1.9
48	*Sanguisorba hakusanensis*	66.4 ± 4.4	57.0 ± 2.0
49	*Euryale ferox*	119.3 ± 3.4	104.0 ± 5.8
50	*Rheum palmatum*	120.4 ± 5.5	79.0 ± 8.5
51	*Rheum undulatum*	108.9 ± 3.2	123.5 ± 4.7
52	*Citrus aurantium*	100.8 ± 1.7	107.5 ± 6.9
53	*Ailanthus altissima*	95.5 ± 6.0	100.1 ± 11.4
54	*Pogostemon cablin*	97.7 ± 2.8	74.4 ± 5.2
55	*Schisandra chinensis*	98.3 ± 2.8	93.5 ± 8.3
56	*Nelumbo nucifera*	101.2 ± 3.5	94.1 ± 5.0
57	*Lindera aggregata*	102.4 ± 5.8	76.5 ± 9.8
58	*Aucklandia lappa*	99.8 ± 3.7	14.7 ± 0.1
59	*Ephedra sinica*	101.7 ± 7.4	79.7 ± 7.7
60	*Fritillaria thunbergii*	103.7 ± 2.6	112.5 ± 7.9
61	*Fritillaria cirrhosa*	111.2 ± 9.4	106.5 ± 6.0
62	*Carex canescens*	101.6 ± 2.1	101.0 ± 10.1
63	*Cinnamomum cassia*	71.5 ± 1.3	32.4 ± 1.7
64	*Piper nigrum*	110.2 ± 4.0	98.0 ± 3.8
65	*Liriope muscari*	100.9 ± 5.5	91.1 ± 5.5
66	*Morus alba*	100.3 ± 5.9	100.2 ± 5.5
67	*Phyllostachys nigra*	97.2 ± 4.6	93.6 ± 2.7
68	*Croton tiglium*	77.4 ± 2.2	64.2 ± 2.1
69	*Houttuynia cordata*	102.1 ± 2.4	107.2 ± 3.3
70	*Perilla frutescens*	102.6 ± 3.4	96.9 ± 2.3
71	*Zanthoxylum piperitum*	92.2 ± 8.4	94.0 ± 7.4

Data are presented as the mean ± SEM.

**Table 2:** NO assay results of the extracts of 1:1 mixtures of two plants.

No.	Plants		% of LPS	
			Water Extract (Group mA)	Ethanol Extract (Group mB)
	Control (0.1% DMSO without LPS)		17.0 ± 0.7	16.5 ± 1.0
1	*Glycyrrhiza uralensis*	*Atractylodes macrocephala*	92.7 ± 3.4	85.9 ± 2.0
2	*Glycyrrhiza uralensis*	*Codonopsis pilosula*	92.3 ± 1.8	83.6 ± 2.2
3	*Glycyrrhiza uralensis*	*Citrus unshiu*	92.8 ± 3.1	82.6 ± 4.6
4	*Glycyrrhiza uralensis*	*Paeonia lactiflora*	96.0 ± 2.6	71.6 ± 4.4
5	*Atractylodes macrocephala*	*Paeonia lactiflora*	91.9 ± 2.6	92.7 ± 4.2
6	*Atractylodes macrocephala*	*Codonopsis pilosula*	90.5 ± 2.1	100.6 ± 2.7
7	*Atractylodes macrocephala*	*Citrus unshiu*	93.7 ± 3.4	90.7 ± 2.6
8	*Paeonia lactiflora*	*Aucklandia lappa*	88.1 ± 5.1	14.8 ± 0.1
9	*Paeonia lactiflora*	*Codonopsis pilosula*	97.0 ± 3.5	92.1 ± 2.8
10	*Paeonia lactiflora*	*Citrus unshiu*	97.2 ± 5.4	93.7 ± 5.1
11	*Astragalus membranaceus*	*Magnolia kobus*	96.7 ± 3.6	77.7 ± 5.8
12	*Astragalus membranaceus*	*Jeffersonia dubia*	75.1 ± 4.4	71.7 ± 2.0
13	*Astragalus membranaceus*	*Aster tataricus*	96.7 ± 4.2	95.2 ± 4.8
14	*Jeffersonia dubia*	*Glycyrrhiza uralensis*	82.8 ± 2.9	71.5 ± 4.6
15	*Jeffersonia dubia*	*Aucklandia lappa*	59.6 ± 2.9	15.9 ± 0.8
16	*Smilax china*	*Rumex japonicus*	104.2 ± 6.2	100.9 ± 6.6
17	*Brassica juncea*	*Pinellia ternata*	93.5 ± 2.5	94.9 ± 4.1
18	*Brassica juncea*	*Zingiber officinale*	94.6 ± 3.0	85.6 ± 3.1
19	*Paeonia lactiflora*	*Jeffersonia dubia*	85.3 ± 2.1	60.7 ± 2.8
20	*Myristica fragrans*	*Aconitum carmichaeli*	92.4 ± 6.2	101.0 ± 1.9
21	*Myristica fragrans*	*Sanguisorba hakusanensis*	68.0 ± 5.8	71.2 ± 1.9
22	*Myristica fragrans*	*Evodia officinalis*	90.9 ± 5.1	91.7 ± 0.8
23	*Myristica fragrans*	*Jeffersonia dubia*	73.2 ± 4.3	69.6 ± 2.4
24	*Myristica fragrans*	*Rheum palmatum*	95.5 ± 7.4	111.9 ± 5.7
25	*Myristica fragrans*	*Psoralea corylifolia*	97.0 ± 6.8	102.8 ± 8.7
26	*Myristica fragrans*	*Zingiber officinale*	118.0 ± 3.0	110.2 ± 4.8
27	*Myristica fragrans*	*Terminalia chebula*	58.4 ± 3.8	51.4 ± 2.4
28	*Myristica fragrans*	*Euryale ferox*	87.2 ± 2.4	88.8 ± 4.4
29	*Myristica fragrans*	*Citrus aurantium*	91.8 ± 11.1	97.4 ± 4.4
30	*Myristica fragrans*	*Machilus thunbergii*	85.9 ± 5.2	92.1 ± 1.9
31	*Myristica fragrans*	*Aucklandia lappa*	79.6 ± 4.5	35.6 ± 1.4
32	*Aconitum carmichaeli*	*Sanguisorba hakusanensis*	66.7 ± 10.2	72.3 ± 6.8
33	*Aconitum carmichaeli*	*Evodia officinalis*	80.1 ± 9.1	84.6 ± 0.0
34	*Aconitum carmichaeli*	*Jeffersonia dubia*	66.5 ± 13.7	68.9 ± 3.1
35	*Aconitum carmichaeli*	*Rheum palmatum*	91.3 ± 12.7	114.7 ± 8.0
36	*Aconitum carmichaeli*	*Psoralea corylifolia*	90.7 ± 0.1	106.4 ± 7.0
37	*Aconitum carmichaeli*	*Zingiber officinale*	94.7 ± 4.4	109.3 ± 1.1
38	*Aconitum carmichaeli*	*Terminalia chebula*	72.5 ± 4.4	49.6 ± 0.9
39	*Aconitum carmichaeli*	*Euryale ferox*	92.3 ± 5.1	101.2 ± 2.6
40	*Aconitum carmichaeli*	*Citrus aurantium*	92.5 ± 6.3	101.2 ± 4.6
41	*Aconitum carmichaeli*	*Machilus thunbergii*	95.6 ± 2.4	101.7 ± 2.4
42	*Aconitum carmichaeli*	*Aucklandia lappa*	85.9 ± 5.9	16.7 ± 1.8
43	*Sanguisorba hakusanensis*	*Evodia officinalis*	82.5 ± 6.9	86.6 ± 2.7
44	*Sanguisorba hakusanensis*	*Jeffersonia dubia*	113.7 ± 3.5	94.2 ± 1.7
45	*Sanguisorba hakusanensis*	*Rheum palmatum*	85.2 ± 12.6	84.4 ± 2.2
46	*Sanguisorba hakusanensis*	*Psoralea corylifolia*	70.4 ± 0.7	76.3 ± 2.7
47	*Sanguisorba hakusanensis*	*Zingiber officinale*	71.7 ± 4.0	63.1 ± 0.9
48	*Sanguisorba hakusanensis*	*Terminalia chebula*	53.1 ± 3.0	52.2 ± 2.4
49	*Sanguisorba hakusanensis*	*Euryale ferox*	60.8 ± 1.0	62.8 ± 1.2
50	*Sanguisorba hakusanensis*	*Citrus aurantium*	72.7 ± 1.3	79.8 ± 2.7
51	*Sanguisorba hakusanensis*	*Machilus thunbergii*	57.2 ± 2.3	79.3 ± 5.2
52	*Sanguisorba hakusanensis*	*Aucklandia lappa*	55.9 ± 2.7	15.4 ± 0.8
53	*Evodia officinalis*	*Jeffersonia dubia*	81.7 ± 3.8	80.3 ± 3.2
54	*Evodia officinalis*	*Rheum palmatum*	112.3 ± 4.7	118.2 ± 3.0
55	*Evodia officinalis*	*Psoralea corylifolia*	102.3 ± 3.2	98.9 ± 1.4
56	*Evodia officinalis*	*Zingiber officinale*	100.8 ± 1.8	98.7 ± 4.1
57	*Evodia officinalis*	*Terminalia chebula*	74.7 ± 1.6	69.5 ± 2.1
58	*Evodia officinalis*	*Euryale ferox*	102.3 ± 2.5	95.6 ± 2.9
59	*Evodia officinalis*	*Citrus aurantium*	100.7 ± 3.0	101.3 ± 3.8
60	*Evodia officinalis*	*Machilus thunbergii*	100.8 ± 3.2	99.1 ± 3.1
61	*Evodia officinalis*	*Aucklandia lappa*	93.6 ± 7.8	34.1 ± 2.3
62	*Jeffersonia dubia*	*Rheum palmatum*	120.1 ± 2.0	106.9 ± 2.3
63	*Jeffersonia dubia*	*Psoralea corylifolia*	100.6 ± 12.6	111.8 ± 1.7
64	*Jeffersonia dubia*	*Zingiber officinale*	92.3 ± 6.1	106.2 ± 4.9
65	*Jeffersonia dubia*	*Terminalia chebula*	86.3 ± 4.7	70.1 ± 2.1
66	*Jeffersonia dubia*	*Euryale ferox*	92.3 ± 3.3	93.5 ± 6.8
67	*Jeffersonia dubia*	*Citrus aurantium*	100.2 ± 4.8	105.3 ± 3.8
68	*Jeffersonia dubia*	*Machilus thunbergii*	101.5 ± 2.1	105.9 ± 4.3
69	*Jeffersonia dubia*	*Aucklandia lappa*	80.4 ± 6.0	25.8 ± 1.7
70	*Rheum palmatum*	*Psoralea corylifolia*	110.5 ± 3.5	118.2 ± 8.7
71	*Rheum palmatum*	*Zingiber officinale*	61.8 ± 8.5	107.6 ± 0.9
72	*Rheum palmatum*	*Terminalia chebula*	105.0 ± 11.4	70.4 ± 5.7
73	*Rheum palmatum*	*Euryale ferox*	113.6 ± 8.8	117.3 ± 4.7
74	*Rheum palmatum*	*Citrus aurantium*	104.0 ± 13.9	107.1 ± 0.2
75	*Rheum palmatum*	*Machilus thunbergii*	109.7 ± 10.9	110.6 ± 2.7
76	*Rheum palmatum*	*Aucklandia lappa*	84.7 ± 5.6	25.3 ± 1.4
77	*Psoralea corylifolia*	*Zingiber officinale*	112.7 ± 16.3	113.2 ± 3.1
78	*Psoralea corylifolia*	*Terminalia chebula*	59.8 ± 4.1	70.0 ± 7.1
79	*Psoralea corylifolia*	*Euryale ferox*	98.3 ± 9.8	114.5 ± 5.2
80	*Psoralea corylifolia*	*Citrus aurantium*	102.9 ± 6.8	109.5 ± 5.2
81	*Psoralea corylifolia*	*Machilus thunbergii*	105.3 ± 2.1	114.5 ± 2.8
82	*Psoralea corylifolia*	*Aucklandia lappa*	93.7 ± 10.1	55.6 ± 2.5
83	*Zingiber officinale*	*Terminalia chebula*	70.2 ± 5.6	62.9 ± 1.3
84	*Zingiber officinale*	*Euryale ferox*	102.6 ± 7.8	107.8 ± 3.6
85	*Zingiber officinale*	*Citrus aurantium*	99.9 ± 4.9	112.2 ± 3.6
86	*Zingiber officinale*	*Machilus thunbergii*	112.6 ± 10.1	109.8 ± 3.7
87	*Zingiber officinale*	*Aucklandia lappa*	99.7 ± 8.4	25.9 ± 2.2
88	*Terminalia chebula*	*Euryale ferox*	59.2 ± 2.5	51.4 ± 2.8
89	*Terminalia chebula*	*Citrus aurantium*	68.5 ± 2.8	60.3 ± 0.9
90	*Terminalia chebula*	*Machilus thunbergii*	59.1 ± 5.5	62.0 ± 6.7
91	*Terminalia chebula*	*Aucklandia lappa*	45.8 ± 2.7	28.0 ± 8.2
92	*Euryale ferox*	*Citrus aurantium*	113.9 ± 14.3	101.7 ± 2.4
93	*Euryale ferox*	*Machilus thunbergii*	118.8 ± 15.1	102.3 ± 7.6
94	*Euryale ferox*	*Aucklandia lappa*	86.2 ± 4.0	24.4 ± 1.4
95	*Citrus aurantium*	*Machilus thunbergii*	108.4 ± 5.6	109.0 ± 7.6
96	*Citrus aurantium*	*Aucklandia lappa*	85.1 ± 3.7	30.1 ± 1.9
97	*Machilus thunbergii*	*Aucklandia lappa*	97.2 ± 5.5	23.9 ± 1.8

Data are presented as the mean ± SEM.

**Table 3:** NO assay results of prescription plant extracts.

No.	Plants		% of LPS	
			Water Extract (Group PA)	Ethanol Extract (Group PB)
	Control (0.1% DMSO without LPS)		22.9 ± 4.8	19.2 ± 0.6
1	*Psoralea corylifolia*	*Myristica fragrans*	100.5 ± 7.3	104.6 ± 3.0
	*Evodia officinalis*	*Schisandra chinensis*		
2	*Paeonia japonica*	*Aucklandia lappa*	95.7 ± 2.3	23.1 ± 1.4
	*Cinnamomum loureirii*	*Cimicifuga heracleifolia*		
3	*Evodia officinalis*	*Paeonia japonica*	95.3 ± 3.3	25.6 ± 1.1
	*Chaenomeles sinensis*	*Aucklandia lappa*		
	*Coix lachryma-jobi*			
4	*Bupleurum falcatum*	*Paeonia japonica*	96.8 ± 2.9	23.2 ± 1.7
	*Cimicifuga heracleifolia*	*Aucklandia lappa*		
5	*Coix lachryma-jobi*	*Amomum villosum*	95.7 ± 8.0	29.6 ±1.7
	*Atractylodes macrocephala*	*Aucklandia lappa*		
6	*Bupleurum falcatum*	*Cimicifuga heracleifolia*	103.8 ± 11.1	106.1 ± 2.1
	*Zingiber officinale*	*Paeonia japonica*		
	*Atractylodes macrocephala*			
7	*Evodia officinalis*	*Aucklandia lappa*	99.1 ± 6.0	46.7 ± 3.2
	*Amomum cardamomum*	*Jeffersonia dubia*		
	*Myristica fragrans*			

Data are presented as the mean ± SEM.

**Table 4:** Inhibitory effects of the selected 72 samples against TNF-α induced adhesion.

Samples	% of TNF-α	Samples	% of TNF-α
Control	45.8 ± 2.8	mA90	103.9 ± 12.7
5-ASA	76.2 ± 1.7	mA91	98.4 ± 5.2
A12	74.8 ± 1.6	mB8	76.6 ± 4.5
A30	61.8 ± 5.9	mB15	94.0 ± 13.1
A36	109.4 ± 8.9	mB19	105.9 ± 3.5
A47	101.4 ± 3.5	mB23	87.2 ± 12.3
A48	106.9 ± 10.8	mB27	103.5 ± 4.1
A63	71.4 ± 5.9	mB31	81.3 ± 9.6
A68	94.1 ± 7.1	mB34	93.1 ± 10.5
B3	74.3 ± 1.5	mB38	96.1 ± 8.3
B12	105.1 ± 6.7	mB42	86.4 ± 5.9
B20	56.5 ± 5.6	mB47	73.2 ± 4.2
B30	53.2 ± 2.4	mB48	98.3 ± 11.7
B32	68.1 ± 5.1	mB49	74.6 ± 7.6
B36	96.7 ± 52	mB52	85.3 ± 6.3
B38	62.0 ± 6.6	mB57	102.9 ± 7.8
B45	74.7 ± 6.5	mB61	88.3 ± 10.9
B47	71.3 ± 6.1	mB65	98.9 ± 9.3
B48	103.9 ± 14.5	mB69	107.8 ± 6.5
B58	53.9 ± 6.2	mB72	95.7 ± 12.6
B63	86.3 ± 11.9	mB76	64.8 ± 4.3
B68	103.7 ± 2.1	mB78	103.5 ± 4.4
mA15	83.0 ± 7.2	mB82	100.3 ± 8.4
mA21	75.0 ± 3.7	mB83	105.5 ± 10.5
mA27	101.3 ± 5.0	mB87	76.2 ± 2.2
mA32	87.2 ± 4.5	mB88	97.6 ± 7.9
mA34	102.3 ± 13.2	mB89	104.1 ± 9.9
mA46	83.7 ± 7.2	mB90	75.4 ± 2.4
mA48	101.9 ± 5.1	mB91	68.9 ± 6.8
mA49	97.3 ± 11.8	mB94	72.5 ± 5.0
mA51	97.6 ± 11.6	mB96	96.6 ± 7.7
mA52	97.9 ± 9.3	mB97	67.4 ± 2.8
mA71	69.9 ± 4.6	PB2	66.5 ± 8.6
mA78	92.5 ± 11.2	PB3	78.9 ± 2.8
mA83	88.9 ± 8.7	PB4	76.2 ± 8.5
mA88	107.5 ± 8.6	PB5	72.9 ± 4.1
mA89	106.7 ± 10.9	PB7	78.7 ± 3.5

Data are presented as the mean ± SEM.
